# Laparoscopic vs Endoscopic Management of Pancreatic Pseudocysts: A Scoping Review

**DOI:** 10.7759/cureus.34694

**Published:** 2023-02-06

**Authors:** Mohim Thakur, Ajay K Dhiman

**Affiliations:** 1 General Surgery, All India Institute of Medical Sciences, Bilaspur, Bilaspur, IND

**Keywords:** pancreas, walled off necrosis, laparoscopy, endoscopy, pancreatic pseudocyst

## Abstract

Pancreatic pseudocyst (PPC) and walled-off necrosis (WON) develop as late complications of acute pancreatitis that have been historically managed surgically. With the advancement in endoscopic equipment and the evolution of endoscopic surgery, the management of PPC has evolved considerably in recent years from surgical drainage to transmural endoscopic drainage. Till the end of the 20th century, a limited number of surgeons performed laparoscopic drainage of PPCs. Due to the steep learning curve needed for performing advanced laparoscopic suturing, a majority of studies conducted during this period have compared open surgical drainage with endoscopy. The efficacy of these modalities has largely been evaluated using retrospective studies and a few meta-analyses particularly due to the low-volume caseload of individual centres. Also, these studies include PPC and WON together in data analysis despite WON being a distinct entity. There are limited prospective well-designed clinical trials comparing endoscopic and laparoscopic management of pure PPCs. There is also a lack of specific recommendations for the management of PPCs. Considerable overlap of indications between these two modalities exists. The efficacy of endoscopic transmural drainage as an index intervention when compared to laparoscopy has not been proven in the research literature. Previous studies have not considered multiple endoscopic interventions within a four-week period of index intervention as a failure. We reviewed the literature using appropriate MeSH terms on the PubMed search engine for articles comparing laparoscopic and endoscopic transmural management of PPCs according to our inclusion and exclusion criteria. Seven articles were identified for inclusion in the qualitative synthesis. This scoping review was conducted to answer some pertinent unanswered questions, identify gaps in knowledge regarding the laparoscopic vs endoscopic management of PPCs, and guide further research.

## Introduction and background

Pancreatic pseudocyst (PPC) is a collection of amylase-rich fluid in the lesser sac encased by a fibrous wall devoid of the epithelium [1]. It is a sequel to both acute and chronic pancreatitis which develops after four weeks of primary insult. It is estimated that 5%-15% of patients with pancreatitis will develop pseudocysts [2]. The primary inciting cause is alcohol intake, cholelithiasis, or trauma. Patients present with symptoms of abdominal pain, early satiety, and nausea, and some may present with a lump in the abdomen [3-4]. Historically, the management of PPC was primarily open surgical internal drainage (cystogastrostomy/jejunostomy). In select patients who were not candidates for general anaesthesia, percutaneous drainage was preferred with an antecedent risk of developing pancreatic fistula, adding to the morbidity. A national comparison was presented in the 2004 Society of Surgery of Alimentary Tract (SSAT). The annual meeting suggests reduced morbidity and mortality after surgical vs percutaneous drainage [5]. With the advent of laparoscopy, in the past two decades, surgeons have become adept at laparoscopic endo-suturing. The development of endo staplers has also helped to further enhance the scope of laparoscopy in the management of PPCs. Laparoscopic internal drainage gives comparable results to open surgery concerning the resolution of cysts, complications, and recurrence on long-term follow-up with the added benefit of decreased postoperative pain and early recovery.

In the last decade, endoscopic transmural drainage with/without endoscopic ultrasound (EUS) guidance has been performed by gastroenterologists in tertiary care hospitals reporting similar results when compared to surgical methods. Research involving a comparison between the laparoscopic and endoscopic approaches in PPCs has largely been restricted to retrospective studies and a few prospective trials. Meta-analysis (of retrospective studies) comparing open surgical treatment with endoscopic drainage has been conducted which shows similar outcomes except for the decreased duration of stay and lower cost burden on the patient in the endoscopy group [6-7]. However, robust prospective randomized studies comparing laparoscopic vs endoscopic drainage of PPC are still scarce. Indications for the management of PPCs with either approach are overlapping, and there is a need to address this dilemma. Most studies, being retrospective in design, are invariably associated with inherent selection bias. Also, studies report multiple endoscopic attempts to achieve the desired outcome and compare it with a single one-stage laparoscopic drainage. There is a need to address these questions by conducting a scoping review of available literature and guiding further research.

Objectives

This article aims to conduct a scoping review of the current state of evidence and also identify knowledge gaps in published literature comparing laparoscopic and endoscopic drainage of PPCs.

## Review

Methods

This scoping review follows the reporting guidelines as outlined in the Preferred Reporting Items for Systematic Reviews and Meta-Analysis extension for Scoping Reviews (PRISMA-Scr) [8].

Search

Relevant articles published (from 2000 to 2022) were searched in databases (PubMed). Studies before the year 2000 were not searched as expertise/management of PPCs has evolved considerably. Research in grey literature was also done by reviewing references on this subject. The following Medical Subject Headings (MeSH) terms were searched in databases: (pancreatic pseudocyst∗ OR pancreatic collections OR pancreatic fluid collections) AND (surgery OR cystogastrostomy OR gastrojejunostomy OR pseudocyst drainage, laparoscopic OR percutaneous drainage OR endosonographic OR surgical drainage OR endoscopic drainage OR endoscopy∗ OR EUS OR endoscopic ultrasound).

Research questions

This review scoped the available literature to respond to the following questions about the subject (endoscopic vs laparoscopic management of pseudocysts). The following questions were addressed in this review: What are the indications for endoscopic and laparoscopic management of PPCs? What is the definition of cyst resolution regarding residual cyst size and timing post-index intervention? What defines failure of intervention? What is the difference in duration of hospital stay? What are the long-term results of the two interventions?

Eligibility criteria

Eligibility criteria were enlisted before the commencement of the study. The aim of a scoping review is not to report all published literature; rather, it is to put forth a snapshot picture of the current status and bring out the gaps in knowledge and give suggestions to improve further research.

Inclusion Criteria

Prospective/retrospective studies, randomized trials and systematic reviews comparing endoscopic and laparoscopic management of PPCs in adult patients (>18 years) published from January 2000 to September 2022 were included.

Exclusion Criteria

All case reports, review (narrative) articles, books and editorials were excluded.

A review protocol was prepared, but since this is not a systematic review, it was not registered in PROSPERO. Studies were reviewed by two independent reviewers. Study characteristics were identified based on population (patients with PPCs), intervention (endoscopic vs laparoscopic drainage of PPC), outcome (resolution of cyst), adverse events (bleeding, infection) and long-term results (recurrence of cyst). Two reviewers independently reviewed articles included in the full-text review, and the inter-rater agreement was 96% (Cohen’s k: 0.89).

Study selection

**Figure 1:**
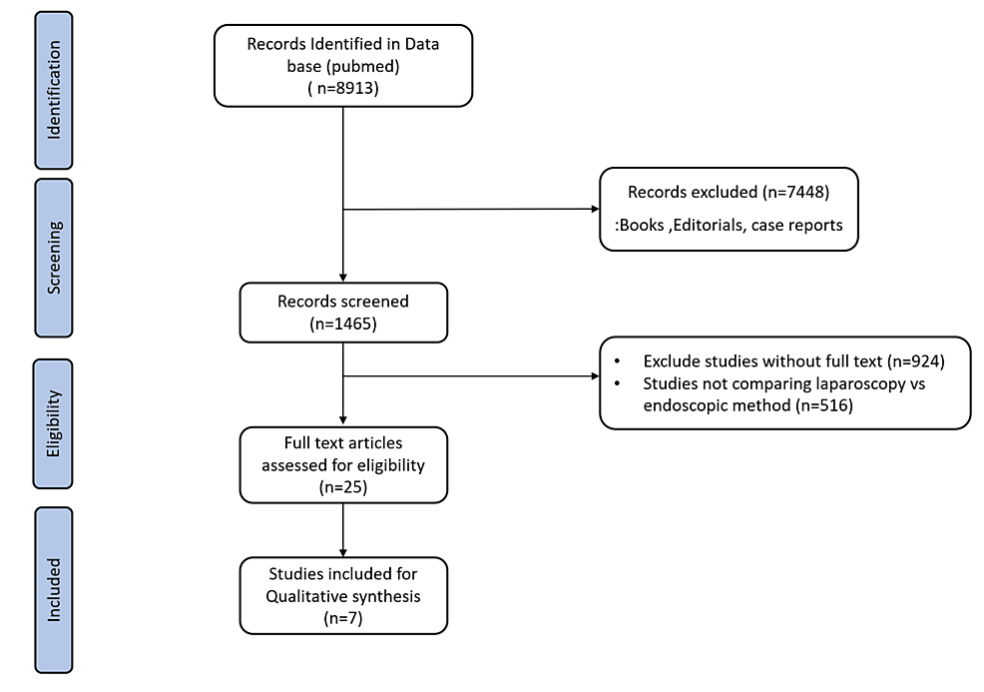
PRISMA flow diagram. PRISMA: Preferred Reporting Items for Systematic Reviews and Meta-Analyses.

A total of 1,465 articles were identified after using the above-mentioned MeSH terms in PubMed search after the exclusion of books, editorials and case reports. Zotero (version 6.09; Corporation for Digital Scholarship, Vienna, United States) was used for reference management. No duplicates were found. After the exclusion of articles without full text and studies not comparing laparoscopy with endoscopic management, 25 full-text articles were shortlisted which were assessed for eligibility. Seven articles were finally included for qualitative synthesis as mentioned in Figure 1.

Results

Indications

There is no consensus on the indications for the endoscopic management of PPCs. In symptomatic mature PPC with a diameter of more than 6 cm which is abutting the stomach and has minimal necrotic debris, it is generally accepted that endoscopic drainage is a suitable treatment [9]. Furthermore, some studies have indicated that endoscopy does not apply to patients with coagulation dysfunction or with multiple PPCs [10]. Surgical drainage is considered more appropriate in symptomatic PPCs with more necrotic debris. The degree of necrotic debris which defines the cut-off for both procedures is not clearly defined. The final decision rests upon individual centre expertise and available infrastructure.

Outcomes

Studies define treatment success as the complete resolution of cyst cavity documented on imaging, measured at four weeks post-index procedure. Reported treatment success rates range from 50% to 100% in endoscopy groups and from 78.9% to 100% in laparoscopic groups. Overall treatment success rates of 89.2% in the endoscopic and 88% in the surgical group have been reported [10]. Therefore, significant differences between the two modalities have not been reported in terms of treatment success in published literature. Data on the long-term success rate of the above interventions are not well documented as most studies have a follow-up period of fewer than two years.

What Defines Failure of Intervention?

Failure of the cyst to resolve after four weeks post-procedure is deemed to be a failure of the intervention. It is important to note that in patients undergoing endoscopic drainage, multiple attempts during these four weeks have not been deemed as a failure. However, a similar analogy has not been applied to laparoscopic drainage due to its relatively more invasive approach.

Complications

Studies have reported adverse events in terms of bleeding and infection. As per a recent meta-analysis, the overall adverse event rate was found to be 11.35% in the endoscopy group and 14.66% in the laparoscopy group [10]. Bleeding was the most common adverse event in the endoscopy group (2.70%), whereas, in the laparoscopy group, it was infection (7.76%). Adverse event rates ranged from 0% to 21.4% in the endoscopy group and from 8.3% to 26.3% in the laparoscopy group. Although this difference was not statistically significant, very few studies report data on the volume of blood loss, which was found to be significantly less in the endoscopy group, probably due to its less invasive nature [11-13]. A multicentre trial by Teoh et al. [14] suggests that the use of a novel self-approximating lumen-apposing metallic stents (LAMS) for the drainage of PPCs was safe and effective. The device was associated with a low rate (6.8%) of stent-related adverse events [14]. Endoscopic necrosectomy in cases of walled-off necrosis (WON) has also been suggested to be feasible. However, patients have to undergo multiple endoscopic procedures for the same [14]. WON is defined by the revised Atlanta classification as a mature, encapsulated collection of pancreatic and/or peripancreatic necrosis and has a well-defined inflammatory wall [15]. However, WON is a distinct entity and should not be clubbed with PPCs for the evaluation of therapeutic interventions.

Duration of Hospital Stay

Few studies have reported data on the length of hospital stay. Comparison between the two groups demonstrated that endoscopic drainage was associated with a significantly shorter length of hospital stay according to previous studies (P = 0.04) [11-13]. Redwan et al. and Varadarajulu et al. have also reported the median length of hospital stay to be significantly shorter in patients who underwent endoscopic cystogastrostomy [16-17]. Recent randomized trials have shown no difference in the median length of hospital stay between the laparoscopic and endoscopic groups using pigtail stents. Criteria for an estimate of the duration of hospital stay in the endoscopic group are not clear in cases undergoing repeated procedures due to failure of index intervention.

Long-Term Results

Recurrence is defined as a new pseudocyst observed by imaging methods at follow-up after a previously reported resolution [7]. Four studies have reported recurrence related to applied therapy. There was no statistical difference found between the two groups regarding recurrence rates [16-19]. Most studies are retrospective in nature with short follow-ups. Therefore, data on long-term recurrence are not available.

Discussion

Management of PPCs has undergone a paradigm shift in the last few decades from conventional open surgery to laparoscopy and now endoscopic techniques. The benefits of endoscopic intervention over surgery are obvious to begin with, particularly due to greater patient acceptance and the less invasive nature of endoscopy. However, these benefits have to be proven in terms of outcomes, adverse events and long-term recurrence rates.

A recent meta-analysis has suggested that there was no difference in the rate of treatment success, adverse events or recurrence between endoscopic and laparoscopic treatment, but the operation time, intraoperative blood loss and hospital stay of endoscopic treatment were significantly less than those of laparoscopic treatment, indicating that endoscopy had certain advantages in PPC treatment [10].

Indications of endoscopic drainage in PPCs are still unclear. The majority suggests that pseudocysts which are large enough and filled with minimal debris/necrotic component (allowing contents to drain freely via a narrow stent) are amenable to endoscopic drainage. Most studies, being retrospective in nature, have a component of selection bias due to the preferential selection of patients with favourable characteristics to a given procedure. Studies have shown that the success rate of endoscopic drainage guided by EUS is more than 90%.

Previous studies reporting similar outcomes between endoscopic and laparoscopic approaches actually needed frequent stent exchanges/repeat procedures in the endoscopic group. In a study by Melman et al. and Li and Qin, 13.3% and 28% of patients underwent repeat endoscopic procedures, respectively [10,13]. Similarly, in a randomized trial by Garg et al., they performed repeated endoscopic intervention in 50% of cases (n=15/30) [20]. Despite repeated procedures, the index intervention was not deemed as a failure in those studies. The above-mentioned studies are comparing multiple endoscopic procedures with single-stage laparoscopic drainage. Patients needing multiple endoscopic interventions should be rather deemed as a failure of the index procedure. This limitation has been countered by authors with the justification that the endoscopic method is a step-up approach by design and repeated procedure cannot be termed as a failure [20]. The relatively non-invasive nature of endoscopic intervention should not justify its repeatability.

There is also no consensus limit on the number of attempts which are considered appropriate for the endoscopic approach. The outcome (complete resolution of cyst cavity) is measured at the end of four weeks post-index procedure in most recent studies, irrespective of the number of repeated attempts during this period. Endoscopy is a relatively less invasive procedure that must not justify its repetition in the event of failure of index intervention. Repetition of any procedure will naturally lead to an escalation of the overall cost burden on the patient. Most recent studies do not take this factor into account. It is worth noting that studies are comparing outcomes for the index endoscopic intervention only despite some patients undergoing repeat procedures. In cases undergoing multiple endoscopic attempts, cumulative duration of hospital stay and blood loss are not taken into account. This four-week period may favour endoscopic drainage such that it is eventually deemed a success in terms of cyst resolution. However, this window period may not be needed in patients undergoing surgical drainage, as cyst resolution can be documented much earlier than four weeks, especially due to a broad stoma which allows complete internal drainage of cyst fluid. The inclusion of WON along with PPCs adds to heterogeneity in data. This can be a possible reason for the need for multiple interventions in an endoscopic group due to inadequate drainage of solid necrotic contents. The follow-up period of studies is mostly up to two years, showing no difference in recurrence rates. However, long-term results are not known due to limited follow-up.

Laparoscopy also gives an additional advantage of managing associated gall stones as a single-step intervention along with cyst drainage when compared to endoscopy. A recent randomized trial by Jagielski et al. suggests that sterile PPCs require no preventive or prophylactic use of antibiotics after endoscopic drainage [21]. The future of endoscopic drainage seems promising in select cases. There is a need to conduct robust randomized trials comparing laparoscopic vs index endoscopic drainage in the management of PPCs with long-term follow-up. Patient treatment should be individualized according to the patient profile, cyst content, size and location.

## Conclusions

Management of PPCs has undergone a paradigm shift. The endoscopic method as a relatively less invasive approach gives comparable outcomes in symptomatic PPCs with minimal necrotic components. Currently, the literature suggests multiple endoscopic drainage attempts as acceptable when compared to single laparoscopic drainage for the management of PPCs. There is a lack of studies evaluating endoscopic transmural drainage as an index procedure. Further robust prospective studies with long-term follow-up are needed to evaluate the outcomes of single-index endoscopic drainage vs laparoscopy in pure PPCs (excluding WON). Laparoscopic drainage of PPC is still an essential tool in the armamentarium of a surgeon when a cyst has a considerable necrotic component, in patients harbouring concomitant gall stones and in low-resource settings where endoscopic interventional facility/expertise is not available. Endoscopy has evolved as a minimally invasive modality for the management of PPCs, which gives comparable outcomes with the additional advantage of a shorter duration of stay and less blood loss when compared to laparoscopy.
